# Crystal structure of poly[[μ_3_-4,4′-(4,4′-bipyridine-2,6-diyl)dibenzoato]{μ_2_-4-[6-(4-carboxyphenyl)-4,4′-bipyridin-4′-ium-2-yl]benzoato}manganese(II)] hemi­hydrate]

**DOI:** 10.1107/S160053681402279X

**Published:** 2014-10-24

**Authors:** Yaping Li, Dajun Sun, Julia Ming, Liying Han, Guanfang Su

**Affiliations:** a Department of Ophthalmology, Yhe Second Hospital of Jilin University, 218 Ziqiang Street, Changchun, 130041, Jilin Province, People’s Republic of China; b Department of Vascular Surgery, The China–Japan Union Hospital of Jilin University, 126 Xiantai Street, Changchun, 130033, Jilin Province, People’s Republic of China; c St Erik’s Eye Hospital, Karolinska Institutet, Polhemsgatan 50, SE-112-82, Stockholm, Sweden; d Department of Gynaecology, Yhe Second Hospital of Jilin University, 218 Ziqiang Street, Changchun, 130041, Jilin Province, People’s Republic of China

**Keywords:** crystal structure, Mn complex, metal-organic coordination polymer, hydrogen bonds

## Abstract

The title compound, {[Mn(C_24_H_14_N_2_O_4_)(C_24_H_16_N_2_O_4_)]·0.5H_2_O}_*n*_, was obtained by the reaction of manganese nitrate with the ligand 4,4′-(4,4′-bi­pyridine-2,6-di­yl) di­benzoic acid under hydro­thermal conditions. The water O atom is located on a twofold rotation axis. The Mn^2+^ ion is hepta­coordinated by six O atoms and one N atom from the ligands. In this structure, the ligands adopts two different forms, one completely deprotonated and one with a protonated N atom (pyridinium) and a carboxylic acid function. In the crystal, N—H⋯O and O—H⋯O hydrogen bonds consolidate the packing, forming a three-dimensional framework.

## Related literature   

For the preparation of the ligand 4,4′-(4,4′-bi­pyridine-2,6-di­yl) di­benzoic acid, see: Hou *et al.* (2010 ▶); Sharma *et al.* (2011 ▶); Song *et al.* (2012 ▶); Wei *et al.* (2013 ▶). For the structures and potential applications of metal-organic coordination polymers involving the 4,4′-(4,4′-bi­pyridine-2,6-di­yl) di­benzoic acid ligand, see: Eddaoudi *et al.* (2002 ▶); Hu *et al.* (2014 ▶); Iremonger *et al.* (2013 ▶).
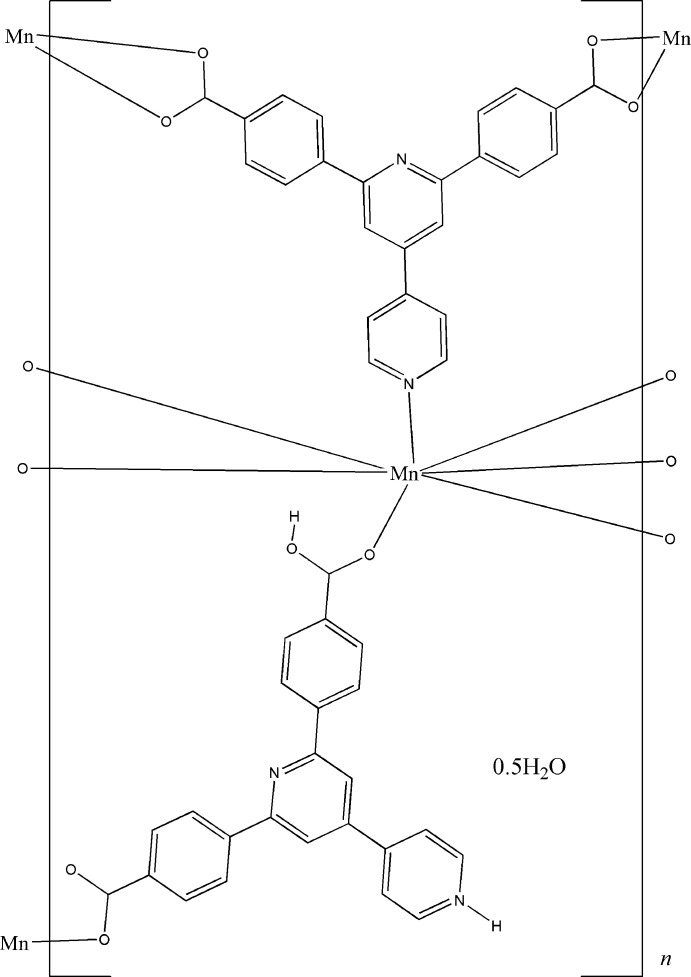


## Experimental   

### Crystal data  

[Mn(C_24_H_14_N_2_O_4_)(C_24_H_16_N_2_O_4_)]·0.5H_2_O
*M* *_r_* = 1709.42
Monoclinic, 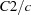

*a* = 26.6396 (13) Å
*b* = 12.9853 (6) Å
*c* = 23.2326 (11) Å
β = 108.696 (1)°
*V* = 7612.6 (6) Å^3^
*Z* = 4
Mo *K*α radiation
μ = 0.42 mm^−1^
*T* = 173 K
0.21 × 0.17 × 0.15 mm

### Data collection  

Bruker APEXII CCD diffractometer
Absorption correction: multi-scan (*SADABS*; Bruker, 2002 ▶) *T*_min_ = 0.918, *T*_max_ = 0.940
20674 measured reflections
7483 independent reflections
5375 reflections with *I* > 2σ(*I*)
*R*_int_ = 0.044

### Refinement  

*R*[*F* ^2^ > 2σ(*F* ^2^)] = 0.061
*wR*(*F* ^2^) = 0.184
*S* = 1.08
7483 reflections
563 parameters
2 restraints
H atoms treated by a mixture of independent and constrained refinement
Δρ_max_ = 0.43 e Å^−3^
Δρ_min_ = −0.73 e Å^−3^

### 

Data collection: *APEX2* (Bruker, 2002 ▶); cell refinement: *SAINT* (Bruker, 2002 ▶); data reduction: *SAINT*; program(s) used to solve structure: *SHELXS97* (Sheldrick, 2008 ▶); program(s) used to refine structure: *SHELXL97* (Sheldrick, 2008 ▶); molecular graphics: *XP* in *SHELXTL* (Sheldrick, 2008 ▶) and *DIAMOND* (Brandenburg, 1999 ▶); software used to prepare material for publication: *SHELXTL* and *publCIF* (Westrip, 2010 ▶).

## Supplementary Material

Crystal structure: contains datablock(s) I, New_Global_Publ_Block. DOI: 10.1107/S160053681402279X/bt6991sup1.cif

**Fig. 1.**
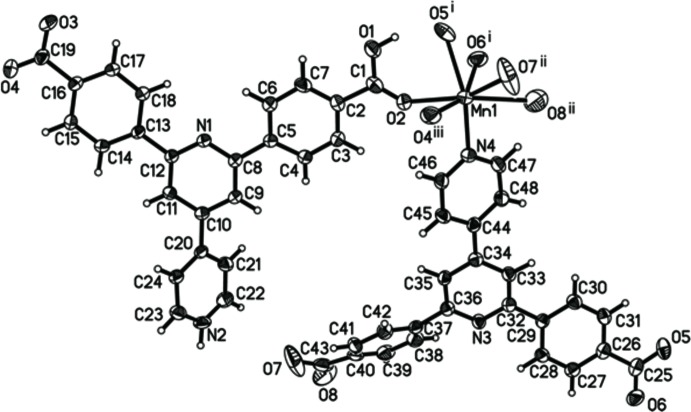
Extended asymmetric unit of the title compound, with the atom-numbering scheme. Displacement ellipsoids are drawn at the 50% probability level. Lattice water molecule has been omitted for clarity. Symmetry codes: i = -0.5 + x,0.5 - y,-0.5 + z; ii = -0.5 + x,0.5 + y,z; iii = x,-1 - y,0.5 + z.

Structure factors: contains datablock(s) I. DOI: 10.1107/S160053681402279X/bt6991Isup2.hkl

x y z x y z x y z . DOI: 10.1107/S160053681402279X/bt6991fig1.tif
Extended asymmetric unit of the title compound, with the atom-numbering scheme. Displacement ellipsoids are drawn at the 50% probability level. Lattice water mol­ecule has been omitted for clarity. Symmetry codes: i = −0.5 + *x*,0.5 − *y*,-0.5 + *z*; ii = −0.5 + *x*,0.5 + *y*,*z*; iii = *x*,-1 − *y*,0.5 + *z*.

**Fig. 2.**
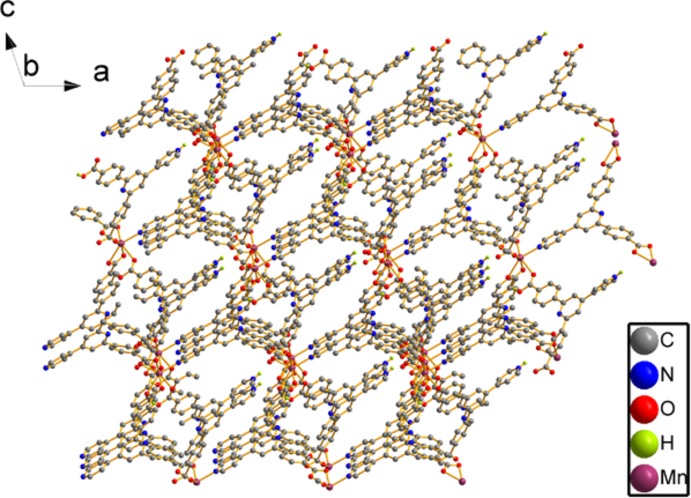
View of the three-dimensional framework of the titled compound.

. DOI: 10.1107/S160053681402279X/bt6991fig2.tif
View of the three-dimensional framework of the titled compound.

CCDC reference: 1029625

Additional supporting information:  crystallographic information; 3D view; checkCIF report

## Figures and Tables

**Table 1:** Hydrogen-bond geometry (, )

*D*H*A*	*D*H	H*A*	*D* *A*	*D*H*A*
N2H2*A*O3^i^	0.89(2)	1.67(2)	2.548(4)	169(5)
O1H1*A*O5^ii^	0.88(2)	1.77(3)	2.612(4)	159(6)
O1*W*H1*W*O3	0.84	2.12	2.963(6)	180

Symmetry codes: (i) 
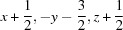
; (ii) 
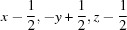
.

## supplementary crystallographic information

### S1. Synthesis and crystallization

A mixture of manganese nitrate aqueous solution (50%, 0.05 mL), 4,4'-(4,4'-bi­pyridine-2,6-diyl) di­benzoic acid (H_2_L 0.0396 g, 0.1 mmol), deionized water (8 mL) and NaOH (aq, 0.1 mol/L, 1 mL) was placed in a 20 mL PTFE-lined stainless steel vessel under autogenous pressure, heated at 160 °C for 72 hours, and allowed to cool down to room temperature in 27 h. The obtained colourless crystals were collected, washed with water and EtOH, and dried under ambient conditions with a yield of 21% based on H_2_L.

### S2. Refinement

All the hydrogen atoms attached to carbon atoms were placed in calculated positions and refined using a riding model. The hydrogen atoms of the protonated carb­oxy­lic group and protonated nitro­gen atom were located from the Fourier difference map. They were refined isotropically with the O—H and N—H distance restrained to 0.89 (2)Å. The water H atom was refined using a riding model.

### S3. Comment

In recent years, much attention has been paid to coordination polymers that have shown perspective in the field of gas adsorption and separation and molecular recognition. Particularly, 4,4'-(4,4'-bipyridine-2,6-diyl) dibenzoic acid was used to form various metal-organic coordination polymers with novel structures (Hou *et al.*, 2010; Sharma *et al.*, 2011; Song *et al.*, 2012, Wei *et al.* 2013). We report here the synthesis and the crystal structure of the title coordination polymer. In the asymmetric unit, there are one crystallographically independent Mn(II) ion, two ligands with different degrees of deprotonation and half a water molecule. The Mn^2+^ ion is heptacoordinated by six O atoms and one nitrogen atom from the ligands (Figure 1). The carboxylate groups of the completely deprotonated *L*^2-^ anion adopt chelating modes, and the nitrogen atom coordinates to the Mn(II) ion; In the other form of the ligand, both the deprotonated carboxylate group and the protonated carboxylic group adopt the monodentate mode, and the nitrogen atom of the terminal pyridyl ring is protonized, not coordinated to the Mn(II) ion. This compound manifests a three-dimensional framework, with versatile hydrogen bonding consolidating the crystal packing (Figure 2).

### Crystal data

**Table:**

[Mn(C_24_H_14_N_2_O_4_)(C_24_H_16_N_2_O_4_)]·0.5H_2_O	*F*(000) = 3520
*M**_r_* = 1709.42	*D*_x_ = 1.491 Mg m^−^^3^
Monoclinic, *C*2/*c*	Mo *K*α radiation, λ = 0.71073 Å
Hall symbol: -C 2yc	Cell parameters from 7485 reflections
*a* = 26.6396 (13) Å	θ = 2.2–25.4°
*b* = 12.9853 (6) Å	µ = 0.42 mm^−^^1^
*c* = 23.2326 (11) Å	*T* = 173 K
β = 108.696 (1)°	Block, colourless
*V* = 7612.6 (6) Å^3^	0.21 × 0.17 × 0.15 mm
*Z* = 4	

### Data collection

**Table:**

Bruker APEXII CCD diffractometer	7483 independent reflections
Radiation source: fine-focus sealed tube	5375 reflections with *I* > 2σ(*I*)
Graphite monochromator	*R*_int_ = 0.044
φ and ω scans	θ_max_ = 26.0°, θ_min_ = 1.6°
Absorption correction: multi-scan (*SADABS*; Bruker, 2002)	*h* = −32→30
*T*_min_ = 0.918, *T*_max_ = 0.940	*k* = −14→16
20674 measured reflections	*l* = −15→28

### Refinement

**Table:**

Refinement on *F*^2^	Primary atom site location: structure-invariant direct methods
Least-squares matrix: full	Secondary atom site location: difference Fourier map
*R*[*F*^2^ > 2σ(*F*^2^)] = 0.061	Hydrogen site location: inferred from neighbouring sites
*wR*(*F*^2^) = 0.184	H atoms treated by a mixture of independent and constrained refinement
*S* = 1.08	*w* = 1/[σ^2^(*F*_o_^2^) + (0.0915*P*)^2^ + 12.2614*P*] where *P* = (*F*_o_^2^ + 2*F*_c_^2^)/3
7483 reflections	(Δ/σ)_max_ = 0.005
563 parameters	Δρ_max_ = 0.43 e Å^−^^3^
2 restraints	Δρ_min_ = −0.73 e Å^−^^3^

### Special details

**Table:**

Geometry. All e.s.d.'s (except the e.s.d. in the dihedral angle between two l.s. planes) are estimated using the full covariance matrix. The cell e.s.d.'s are taken into account individually in the estimation of e.s.d.'s in distances, angles and torsion angles; correlations between e.s.d.'s in cell parameters are only used when they are defined by crystal symmetry. An approximate (isotropic) treatment of cell e.s.d.'s is used for estimating e.s.d.'s involving l.s. planes.
Refinement. Refinement of *F*^2^ against ALL reflections. The weighted *R*-factor *wR* and goodness of fit *S* are based on *F*^2^, conventional *R*-factors *R* are based on *F*, with *F* set to zero for negative *F*^2^. The threshold expression of *F*^2^ > σ(*F*^2^) is used only for calculating *R*-factors(gt) *etc*. and is not relevant to the choice of reflections for refinement. *R*-factors based on *F*^2^ are statistically about twice as large as those based on *F*, and *R*- factors based on ALL data will be even larger.

### Fractional atomic coordinates and isotropic or equivalent isotropic displacement parameters (Å^2^)

**Table:**

	*x*	*y*	*z*	*U*_iso_*/*U*_eq_	
C1	0.15804 (14)	−0.1097 (3)	0.08907 (18)	0.0344 (8)	
C2	0.18395 (13)	−0.1986 (3)	0.06874 (16)	0.0293 (7)	
C3	0.23710 (14)	−0.2152 (3)	0.09876 (16)	0.0326 (8)	
H3	0.2562	−0.1694	0.1300	0.039*	
C4	0.26243 (14)	−0.2981 (3)	0.08354 (16)	0.0318 (8)	
H4	0.2991	−0.3081	0.1042	0.038*	
C5	0.23562 (13)	−0.3677 (3)	0.03863 (15)	0.0266 (7)	
C6	0.18188 (14)	−0.3499 (3)	0.00811 (17)	0.0338 (8)	
H6	0.1627	−0.3962	−0.0228	0.041*	
C7	0.15633 (13)	−0.2654 (3)	0.02249 (17)	0.0340 (8)	
H7	0.1200	−0.2531	0.0008	0.041*	
C8	0.26298 (13)	−0.4577 (2)	0.02238 (15)	0.0269 (7)	
C9	0.31506 (13)	−0.4836 (3)	0.05586 (15)	0.0287 (7)	
H9	0.3344	−0.4435	0.0900	0.034*	
C10	0.33808 (12)	−0.5687 (3)	0.03856 (15)	0.0269 (7)	
C11	0.30880 (12)	−0.6252 (3)	−0.01208 (15)	0.0272 (7)	
H11	0.3238	−0.6835	−0.0251	0.033*	
C12	0.25691 (12)	−0.5943 (2)	−0.04327 (15)	0.0258 (7)	
C13	0.22326 (12)	−0.6493 (3)	−0.09783 (15)	0.0263 (7)	
C14	0.23137 (13)	−0.7519 (3)	−0.10911 (16)	0.0312 (8)	
H14	0.2599	−0.7886	−0.0815	0.037*	
C15	0.19837 (13)	−0.8015 (3)	−0.16011 (15)	0.0299 (8)	
H15	0.2048	−0.8714	−0.1676	0.036*	
C16	0.15599 (12)	−0.7494 (3)	−0.20014 (15)	0.0272 (7)	
C17	0.14738 (13)	−0.6464 (3)	−0.18881 (15)	0.0289 (8)	
H17	0.1182	−0.6102	−0.2157	0.035*	
C18	0.18100 (13)	−0.5974 (3)	−0.13884 (16)	0.0305 (8)	
H18	0.1753	−0.5268	−0.1321	0.037*	
C19	0.11957 (13)	−0.8022 (3)	−0.25523 (16)	0.0313 (8)	
C20	0.39234 (13)	−0.6008 (3)	0.07505 (15)	0.0274 (7)	
C21	0.43358 (14)	−0.5290 (3)	0.09350 (18)	0.0362 (9)	
H21	0.4277	−0.4592	0.0809	0.043*	
C22	0.48295 (14)	−0.5609 (3)	0.13019 (18)	0.0392 (9)	
H22	0.5113	−0.5130	0.1425	0.047*	
C23	0.45268 (14)	−0.7284 (3)	0.13116 (16)	0.0345 (8)	
H23	0.4598	−0.7974	0.1450	0.041*	
C24	0.40293 (13)	−0.7023 (3)	0.09369 (15)	0.0296 (8)	
H24	0.3761	−0.7532	0.0807	0.036*	
C25	0.60244 (15)	0.3586 (3)	0.60941 (17)	0.0338 (8)	
C26	0.57244 (14)	0.2860 (3)	0.56016 (16)	0.0317 (8)	
C27	0.59484 (14)	0.1928 (3)	0.55209 (16)	0.0318 (8)	
H27	0.6298	0.1761	0.5769	0.038*	
C28	0.56647 (13)	0.1237 (3)	0.50800 (16)	0.0301 (8)	
H28	0.5823	0.0604	0.5027	0.036*	
C29	0.51525 (13)	0.1466 (3)	0.47165 (15)	0.0265 (7)	
C30	0.49341 (14)	0.2411 (3)	0.47997 (16)	0.0319 (8)	
H30	0.4584	0.2580	0.4553	0.038*	
C31	0.52148 (14)	0.3096 (3)	0.52296 (16)	0.0322 (8)	
H31	0.5060	0.3738	0.5274	0.039*	
C32	0.48572 (13)	0.0733 (3)	0.42392 (15)	0.0269 (7)	
C33	0.43125 (13)	0.0773 (3)	0.39712 (15)	0.0285 (7)	
H33	0.4112	0.1278	0.4096	0.034*	
C34	0.40595 (13)	0.0070 (3)	0.35177 (15)	0.0268 (7)	
C35	0.43732 (13)	−0.0673 (3)	0.33699 (15)	0.0280 (7)	
H35	0.4216	−0.1176	0.3069	0.034*	
C36	0.49143 (13)	−0.0679 (3)	0.36618 (15)	0.0274 (7)	
C37	0.52608 (13)	−0.1435 (3)	0.34994 (15)	0.0292 (8)	
C38	0.57489 (14)	−0.1129 (3)	0.34588 (17)	0.0399 (9)	
H38	0.5876	−0.0453	0.3578	0.048*	
C39	0.60491 (14)	−0.1801 (4)	0.32468 (17)	0.0444 (10)	
H39	0.6380	−0.1580	0.3216	0.053*	
C40	0.58758 (16)	−0.2789 (3)	0.30785 (16)	0.0427 (10)	
C41	0.54046 (17)	−0.3112 (3)	0.31473 (17)	0.0414 (10)	
H41	0.5291	−0.3803	0.3052	0.050*	
C42	0.50987 (15)	−0.2447 (3)	0.33512 (16)	0.0361 (8)	
H42	0.4773	−0.2679	0.3392	0.043*	
C43	0.6166 (2)	−0.3465 (5)	0.27726 (19)	0.0629 (15)	
C44	0.34825 (13)	0.0117 (3)	0.32027 (15)	0.0273 (7)	
C45	0.31961 (13)	−0.0753 (3)	0.29198 (16)	0.0316 (8)	
H45	0.3368	−0.1400	0.2943	0.038*	
C46	0.26630 (13)	−0.0657 (3)	0.26079 (17)	0.0323 (8)	
H46	0.2475	−0.1252	0.2418	0.039*	
C47	0.26692 (14)	0.1056 (3)	0.28296 (17)	0.0349 (8)	
H47	0.2486	0.1691	0.2799	0.042*	
C48	0.32012 (13)	0.1033 (3)	0.31498 (16)	0.0314 (8)	
H48	0.3377	0.1643	0.3335	0.038*	
N1	0.23494 (10)	−0.5126 (2)	−0.02606 (12)	0.0272 (6)	
N2	0.49093 (11)	−0.6582 (3)	0.14826 (14)	0.0360 (7)	
H2A	0.5220 (11)	−0.683 (4)	0.1709 (19)	0.072 (15)*	
N3	0.51581 (11)	0.0015 (2)	0.40859 (13)	0.0284 (6)	
N4	0.23962 (11)	0.0226 (2)	0.25578 (13)	0.0309 (7)	
O1W	0.0000	−0.6159 (6)	−0.2500	0.152 (3)	
H1W	0.0212	−0.6573	−0.2578	0.228*	
O1	0.10826 (10)	−0.0956 (2)	0.05641 (13)	0.0434 (7)	
H1A	0.093 (2)	−0.042 (3)	0.067 (3)	0.10 (2)*	
O2	0.18203 (10)	−0.0563 (2)	0.13208 (12)	0.0422 (7)	
O3	0.07424 (10)	−0.7629 (2)	−0.27753 (14)	0.0529 (8)	
O4	0.13619 (11)	−0.8777 (2)	−0.27658 (12)	0.0443 (7)	
O5	0.58121 (10)	0.44425 (19)	0.61444 (13)	0.0424 (7)	
O6	0.64591 (12)	0.3330 (2)	0.64400 (13)	0.0508 (8)	
O7	0.5949 (2)	−0.4275 (3)	0.2519 (2)	0.1033 (16)	
O8	0.65938 (15)	−0.3180 (4)	0.27336 (17)	0.1032 (17)	
Mn1	0.15272 (2)	0.03181 (4)	0.20581 (2)	0.03203 (18)	

### Atomic displacement parameters (Å^2^)

**Table:**

	*U*^11^	*U*^22^	*U*^33^	*U*^12^	*U*^13^	*U*^23^
C1	0.0324 (19)	0.0280 (19)	0.044 (2)	0.0034 (15)	0.0143 (17)	0.0026 (17)
C2	0.0343 (18)	0.0250 (17)	0.0314 (18)	0.0023 (14)	0.0145 (16)	0.0003 (15)
C3	0.0362 (19)	0.0307 (19)	0.0272 (18)	0.0016 (15)	0.0049 (15)	−0.0042 (15)
C4	0.0271 (17)	0.0338 (19)	0.0310 (18)	0.0062 (15)	0.0046 (15)	−0.0047 (16)
C5	0.0242 (16)	0.0286 (18)	0.0247 (17)	0.0034 (13)	0.0046 (14)	−0.0010 (14)
C6	0.0294 (18)	0.0320 (19)	0.035 (2)	0.0013 (15)	0.0030 (16)	−0.0069 (16)
C7	0.0240 (17)	0.036 (2)	0.040 (2)	0.0056 (15)	0.0083 (15)	0.0005 (17)
C8	0.0255 (17)	0.0280 (18)	0.0248 (17)	−0.0011 (14)	0.0046 (14)	−0.0027 (14)
C9	0.0264 (17)	0.0289 (18)	0.0259 (17)	0.0013 (14)	0.0013 (14)	−0.0040 (15)
C10	0.0214 (16)	0.0293 (17)	0.0274 (18)	0.0009 (13)	0.0041 (14)	−0.0010 (15)
C11	0.0213 (16)	0.0293 (18)	0.0292 (18)	0.0049 (13)	0.0057 (14)	−0.0039 (15)
C12	0.0226 (16)	0.0270 (17)	0.0258 (17)	0.0006 (13)	0.0050 (14)	−0.0030 (14)
C13	0.0202 (15)	0.0289 (18)	0.0263 (17)	−0.0009 (13)	0.0024 (13)	−0.0047 (14)
C14	0.0241 (17)	0.0330 (19)	0.0313 (19)	0.0062 (14)	0.0014 (15)	−0.0030 (16)
C15	0.0292 (18)	0.0270 (18)	0.0306 (18)	−0.0001 (14)	0.0058 (15)	−0.0072 (15)
C16	0.0236 (16)	0.0309 (18)	0.0243 (17)	−0.0031 (14)	0.0038 (14)	−0.0030 (14)
C17	0.0237 (16)	0.0302 (18)	0.0276 (18)	0.0015 (14)	0.0010 (14)	−0.0007 (15)
C18	0.0259 (17)	0.0283 (18)	0.0320 (19)	0.0012 (14)	0.0018 (15)	−0.0041 (15)
C19	0.0289 (18)	0.0287 (19)	0.0330 (19)	−0.0078 (15)	0.0050 (15)	−0.0027 (16)
C20	0.0227 (16)	0.0336 (19)	0.0225 (16)	0.0035 (14)	0.0026 (13)	−0.0056 (14)
C21	0.0274 (18)	0.0311 (19)	0.044 (2)	0.0019 (15)	0.0023 (16)	−0.0023 (17)
C22	0.0261 (18)	0.043 (2)	0.042 (2)	−0.0049 (16)	0.0024 (17)	−0.0054 (18)
C23	0.0318 (19)	0.037 (2)	0.0338 (19)	0.0085 (16)	0.0086 (16)	0.0018 (16)
C24	0.0244 (17)	0.0329 (19)	0.0284 (18)	0.0016 (14)	0.0040 (14)	−0.0038 (15)
C25	0.044 (2)	0.029 (2)	0.0313 (19)	−0.0137 (16)	0.0172 (18)	−0.0035 (16)
C26	0.041 (2)	0.0253 (18)	0.0304 (19)	−0.0086 (15)	0.0140 (16)	−0.0005 (15)
C27	0.0335 (19)	0.0294 (19)	0.0308 (19)	−0.0025 (15)	0.0079 (15)	0.0021 (15)
C28	0.0326 (18)	0.0244 (17)	0.0318 (19)	0.0007 (14)	0.0080 (15)	0.0012 (15)
C29	0.0273 (17)	0.0256 (17)	0.0264 (17)	−0.0030 (13)	0.0084 (14)	−0.0016 (14)
C30	0.0272 (17)	0.0319 (19)	0.036 (2)	0.0003 (15)	0.0100 (15)	−0.0010 (16)
C31	0.0366 (19)	0.0257 (18)	0.037 (2)	−0.0009 (15)	0.0154 (17)	−0.0017 (15)
C32	0.0280 (17)	0.0246 (17)	0.0288 (18)	−0.0025 (14)	0.0099 (14)	0.0005 (14)
C33	0.0271 (17)	0.0282 (18)	0.0306 (18)	0.0019 (14)	0.0098 (15)	−0.0024 (15)
C34	0.0239 (16)	0.0298 (18)	0.0280 (17)	−0.0002 (14)	0.0101 (14)	0.0007 (14)
C35	0.0271 (17)	0.0312 (18)	0.0257 (17)	0.0008 (14)	0.0084 (14)	−0.0048 (15)
C36	0.0276 (17)	0.0306 (18)	0.0264 (17)	−0.0001 (14)	0.0120 (14)	0.0000 (15)
C37	0.0241 (17)	0.037 (2)	0.0244 (17)	0.0042 (14)	0.0054 (14)	−0.0029 (15)
C38	0.0272 (19)	0.050 (2)	0.040 (2)	0.0027 (16)	0.0085 (17)	−0.0037 (19)
C39	0.0243 (18)	0.074 (3)	0.035 (2)	0.0143 (19)	0.0096 (16)	−0.002 (2)
C40	0.045 (2)	0.056 (3)	0.0226 (18)	0.032 (2)	0.0046 (17)	0.0037 (18)
C41	0.059 (3)	0.033 (2)	0.031 (2)	0.0160 (18)	0.0136 (19)	0.0010 (17)
C42	0.040 (2)	0.039 (2)	0.0318 (19)	0.0010 (16)	0.0154 (17)	−0.0003 (17)
C43	0.066 (3)	0.089 (4)	0.032 (2)	0.051 (3)	0.012 (2)	0.008 (2)
C44	0.0265 (17)	0.0320 (18)	0.0245 (17)	0.0004 (14)	0.0098 (14)	−0.0038 (15)
C45	0.0247 (17)	0.0300 (18)	0.039 (2)	0.0043 (14)	0.0080 (15)	−0.0021 (16)
C46	0.0284 (18)	0.0307 (19)	0.036 (2)	−0.0004 (15)	0.0079 (16)	−0.0075 (16)
C47	0.0307 (19)	0.0295 (19)	0.043 (2)	0.0057 (15)	0.0104 (17)	−0.0052 (17)
C48	0.0238 (17)	0.0345 (19)	0.036 (2)	−0.0018 (14)	0.0095 (15)	−0.0071 (16)
N1	0.0235 (14)	0.0294 (15)	0.0254 (15)	0.0022 (12)	0.0031 (12)	−0.0025 (12)
N2	0.0221 (15)	0.048 (2)	0.0312 (16)	0.0080 (14)	−0.0010 (13)	0.0013 (15)
N3	0.0259 (14)	0.0283 (15)	0.0315 (15)	0.0000 (12)	0.0098 (13)	−0.0038 (13)
N4	0.0234 (14)	0.0346 (17)	0.0318 (16)	0.0040 (12)	0.0047 (12)	−0.0031 (13)
O1W	0.149 (7)	0.129 (6)	0.172 (8)	0.000	0.042 (6)	0.000
O1	0.0311 (14)	0.0365 (15)	0.0582 (18)	0.0046 (12)	0.0081 (13)	−0.0120 (14)
O2	0.0382 (15)	0.0433 (15)	0.0389 (15)	0.0106 (12)	0.0038 (12)	−0.0126 (13)
O3	0.0278 (14)	0.0532 (18)	0.0603 (19)	−0.0012 (12)	−0.0104 (13)	−0.0183 (15)
O4	0.0503 (16)	0.0330 (14)	0.0421 (16)	−0.0022 (12)	0.0041 (13)	−0.0109 (13)
O5	0.0425 (15)	0.0308 (14)	0.0606 (19)	−0.0135 (12)	0.0258 (14)	−0.0171 (13)
O6	0.0585 (19)	0.0368 (15)	0.0405 (16)	−0.0039 (14)	−0.0073 (14)	−0.0072 (13)
O7	0.166 (5)	0.054 (2)	0.130 (4)	0.035 (3)	0.103 (4)	0.001 (3)
O8	0.051 (2)	0.186 (5)	0.067 (2)	0.044 (3)	0.0108 (19)	−0.044 (3)
Mn1	0.0287 (3)	0.0319 (3)	0.0309 (3)	0.0070 (2)	0.0030 (2)	0.0009 (2)

### Geometric parameters (Å, º)

**Table:**

C1—O2	1.216 (4)	C28—H28	0.9500
C1—O1	1.312 (4)	C29—C30	1.397 (5)
C1—C2	1.497 (5)	C29—C32	1.482 (5)
C2—C3	1.380 (5)	C30—C31	1.368 (5)
C2—C7	1.394 (5)	C30—H30	0.9500
C3—C4	1.376 (5)	C31—H31	0.9500
C3—H3	0.9500	C32—N3	1.349 (4)
C4—C5	1.392 (5)	C32—C33	1.384 (5)
C4—H4	0.9500	C33—C34	1.393 (5)
C5—C6	1.398 (5)	C33—H33	0.9500
C5—C8	1.489 (5)	C34—C35	1.390 (5)
C6—C7	1.387 (5)	C34—C44	1.477 (5)
C6—H6	0.9500	C35—C36	1.383 (5)
C7—H7	0.9500	C35—H35	0.9500
C8—N1	1.339 (4)	C36—N3	1.339 (4)
C8—C9	1.397 (5)	C36—C37	1.477 (5)
C9—C10	1.384 (5)	C37—C38	1.391 (5)
C9—H9	0.9500	C37—C42	1.392 (5)
C10—C11	1.394 (5)	C38—C39	1.376 (5)
C10—C20	1.482 (4)	C38—H38	0.9500
C11—C12	1.398 (4)	C39—C40	1.378 (6)
C11—H11	0.9500	C39—H39	0.9500
C12—N1	1.334 (4)	C40—C41	1.380 (6)
C12—C13	1.481 (4)	C40—C43	1.491 (6)
C13—C14	1.387 (5)	C41—C42	1.372 (5)
C13—C18	1.394 (5)	C41—H41	0.9500
C14—C15	1.387 (5)	C42—H42	0.9500
C14—H14	0.9500	C43—O8	1.230 (7)
C15—C16	1.387 (5)	C43—O7	1.253 (7)
C15—H15	0.9500	C44—C48	1.390 (5)
C16—C17	1.395 (5)	C44—C45	1.403 (5)
C16—C19	1.502 (5)	C45—C46	1.378 (5)
C17—C18	1.374 (5)	C45—H45	0.9500
C17—H17	0.9500	C46—N4	1.334 (4)
C18—H18	0.9500	C46—H46	0.9500
C19—O4	1.242 (4)	C47—N4	1.339 (4)
C19—O3	1.260 (4)	C47—C48	1.374 (5)
C20—C24	1.387 (5)	C47—H47	0.9500
C20—C21	1.400 (5)	C48—H48	0.9500
C21—C22	1.382 (5)	N2—H2A	0.885 (19)
C21—H21	0.9500	N4—Mn1	2.236 (3)
C22—N2	1.326 (5)	O1W—H1W	0.8398
C22—H22	0.9500	O1—H1A	0.88 (2)
C23—N2	1.330 (5)	O2—Mn1	2.390 (3)
C23—C24	1.375 (5)	O4—Mn1^ii^	2.117 (3)
C23—H23	0.9500	O5—Mn1^i^	2.375 (3)
C24—H24	0.9500	O6—Mn1^i^	2.238 (3)
C25—O6	1.225 (5)	O7—Mn1^iii^	2.202 (4)
C25—O5	1.270 (4)	O8—Mn1^iii^	2.474 (4)
C25—C26	1.499 (5)	Mn1—O4^iv^	2.117 (3)
C25—Mn1^i^	2.629 (4)	Mn1—O7^v^	2.202 (4)
C26—C31	1.390 (5)	Mn1—O6^vi^	2.238 (3)
C26—C27	1.389 (5)	Mn1—O5^vi^	2.375 (3)
C27—C28	1.388 (5)	Mn1—O8^v^	2.474 (4)
C27—H27	0.9500	Mn1—C25^vi^	2.629 (4)
C28—C29	1.387 (5)		
			
O2—C1—O1	124.3 (3)	C26—C31—H31	119.8
O2—C1—C2	121.7 (3)	N3—C32—C33	122.2 (3)
O1—C1—C2	114.1 (3)	N3—C32—C29	115.1 (3)
C3—C2—C7	119.7 (3)	C33—C32—C29	122.7 (3)
C3—C2—C1	117.6 (3)	C32—C33—C34	119.9 (3)
C7—C2—C1	122.7 (3)	C32—C33—H33	120.0
C4—C3—C2	120.1 (3)	C34—C33—H33	120.0
C4—C3—H3	120.0	C35—C34—C33	117.3 (3)
C2—C3—H3	120.0	C35—C34—C44	121.3 (3)
C3—C4—C5	121.6 (3)	C33—C34—C44	121.5 (3)
C3—C4—H4	119.2	C36—C35—C34	119.8 (3)
C5—C4—H4	119.2	C36—C35—H35	120.1
C4—C5—C6	118.0 (3)	C34—C35—H35	120.1
C4—C5—C8	121.6 (3)	N3—C36—C35	122.7 (3)
C6—C5—C8	120.4 (3)	N3—C36—C37	116.1 (3)
C7—C6—C5	120.7 (3)	C35—C36—C37	121.1 (3)
C7—C6—H6	119.7	C38—C37—C42	118.4 (3)
C5—C6—H6	119.7	C38—C37—C36	120.1 (3)
C6—C7—C2	120.0 (3)	C42—C37—C36	121.4 (3)
C6—C7—H7	120.0	C39—C38—C37	120.4 (4)
C2—C7—H7	120.0	C39—C38—H38	119.8
N1—C8—C9	121.8 (3)	C37—C38—H38	119.8
N1—C8—C5	116.4 (3)	C38—C39—C40	120.7 (4)
C9—C8—C5	121.9 (3)	C38—C39—H39	119.6
C10—C9—C8	119.0 (3)	C40—C39—H39	119.6
C10—C9—H9	120.5	C39—C40—C41	119.0 (3)
C8—C9—H9	120.5	C39—C40—C43	120.4 (4)
C9—C10—C11	119.0 (3)	C41—C40—C43	120.4 (4)
C9—C10—C20	120.0 (3)	C42—C41—C40	120.8 (4)
C11—C10—C20	120.9 (3)	C42—C41—H41	119.6
C10—C11—C12	118.6 (3)	C40—C41—H41	119.6
C10—C11—H11	120.7	C41—C42—C37	120.6 (4)
C12—C11—H11	120.7	C41—C42—H42	119.7
N1—C12—C11	121.9 (3)	C37—C42—H42	119.7
N1—C12—C13	115.9 (3)	O8—C43—O7	121.0 (5)
C11—C12—C13	122.1 (3)	O8—C43—C40	119.5 (6)
C14—C13—C18	118.4 (3)	O7—C43—C40	119.2 (5)
C14—C13—C12	122.5 (3)	C48—C44—C45	116.8 (3)
C18—C13—C12	119.1 (3)	C48—C44—C34	121.7 (3)
C13—C14—C15	120.9 (3)	C45—C44—C34	121.5 (3)
C13—C14—H14	119.6	C46—C45—C44	119.2 (3)
C15—C14—H14	119.6	C46—C45—H45	120.4
C16—C15—C14	120.2 (3)	C44—C45—H45	120.4
C16—C15—H15	119.9	N4—C46—C45	123.7 (3)
C14—C15—H15	119.9	N4—C46—H46	118.2
C15—C16—C17	119.2 (3)	C45—C46—H46	118.2
C15—C16—C19	120.8 (3)	N4—C47—C48	123.2 (3)
C17—C16—C19	119.9 (3)	N4—C47—H47	118.4
C18—C17—C16	120.1 (3)	C48—C47—H47	118.4
C18—C17—H17	120.0	C47—C48—C44	120.0 (3)
C16—C17—H17	120.0	C47—C48—H48	120.0
C17—C18—C13	121.2 (3)	C44—C48—H48	120.0
C17—C18—H18	119.4	C12—N1—C8	119.7 (3)
C13—C18—H18	119.4	C22—N2—C23	121.9 (3)
O4—C19—O3	124.9 (3)	C22—N2—H2A	124 (3)
O4—C19—C16	119.1 (3)	C23—N2—H2A	114 (3)
O3—C19—C16	116.0 (3)	C36—N3—C32	118.0 (3)
C24—C20—C21	118.4 (3)	C46—N4—C47	117.2 (3)
C24—C20—C10	120.6 (3)	C46—N4—Mn1	121.7 (2)
C21—C20—C10	120.9 (3)	C47—N4—Mn1	121.2 (2)
C22—C21—C20	119.2 (3)	C1—O1—H1A	114 (4)
C22—C21—H21	120.4	C1—O2—Mn1	131.0 (2)
C20—C21—H21	120.4	C19—O4—Mn1^ii^	161.0 (3)
N2—C22—C21	120.4 (3)	C25—O5—Mn1^i^	86.8 (2)
N2—C22—H22	119.8	C25—O6—Mn1^i^	94.2 (2)
C21—C22—H22	119.8	C43—O7—Mn1^iii^	98.2 (4)
N2—C23—C24	120.7 (3)	C43—O8—Mn1^iii^	86.0 (3)
N2—C23—H23	119.6	O4^iv^—Mn1—O7^v^	84.95 (14)
C24—C23—H23	119.6	O4^iv^—Mn1—N4	95.40 (11)
C23—C24—C20	119.4 (3)	O7^v^—Mn1—N4	121.96 (17)
C23—C24—H24	120.3	O4^iv^—Mn1—O6^vi^	152.41 (11)
C20—C24—H24	120.3	O7^v^—Mn1—O6^vi^	101.41 (13)
O6—C25—O5	122.1 (3)	N4—Mn1—O6^vi^	103.44 (11)
O6—C25—C26	119.4 (3)	O4^iv^—Mn1—O5^vi^	98.02 (10)
O5—C25—C26	118.4 (3)	O7^v^—Mn1—O5^vi^	85.26 (16)
O6—C25—Mn1^i^	58.08 (19)	N4—Mn1—O5^vi^	150.64 (10)
O5—C25—Mn1^i^	64.38 (19)	O6^vi^—Mn1—O5^vi^	56.42 (9)
C26—C25—Mn1^i^	172.5 (2)	O4^iv^—Mn1—O2	79.75 (11)
C31—C26—C27	119.0 (3)	O7^v^—Mn1—O2	154.57 (16)
C31—C26—C25	120.8 (3)	N4—Mn1—O2	79.91 (10)
C27—C26—C25	120.2 (3)	O6^vi^—Mn1—O2	83.88 (11)
C28—C27—C26	120.4 (3)	O5^vi^—Mn1—O2	76.98 (9)
C28—C27—H27	119.8	O4^iv^—Mn1—O8^v^	126.86 (15)
C26—C27—H27	119.8	O7^v^—Mn1—O8^v^	54.68 (16)
C29—C28—C27	120.5 (3)	N4—Mn1—O8^v^	82.01 (11)
C29—C28—H28	119.8	O6^vi^—Mn1—O8^v^	76.30 (14)
C27—C28—H28	119.8	O5^vi^—Mn1—O8^v^	109.74 (11)
C28—C29—C30	118.4 (3)	O2—Mn1—O8^v^	149.17 (13)
C28—C29—C32	120.0 (3)	O4^iv^—Mn1—C25^vi^	126.56 (11)
C30—C29—C32	121.5 (3)	O7^v^—Mn1—C25^vi^	92.25 (15)
C31—C30—C29	121.2 (3)	N4—Mn1—C25^vi^	129.14 (12)
C31—C30—H30	119.4	O6^vi^—Mn1—C25^vi^	27.68 (11)
C29—C30—H30	119.4	O5^vi^—Mn1—C25^vi^	28.84 (10)
C30—C31—C26	120.5 (3)	O2—Mn1—C25^vi^	80.96 (10)
C30—C31—H31	119.8	O8^v^—Mn1—C25^vi^	91.53 (13)
			
O2—C1—C2—C3	−3.1 (5)	C34—C35—C36—N3	−0.5 (5)
O1—C1—C2—C3	176.7 (3)	C34—C35—C36—C37	−177.7 (3)
O2—C1—C2—C7	175.2 (3)	N3—C36—C37—C38	−38.7 (5)
O1—C1—C2—C7	−5.1 (5)	C35—C36—C37—C38	138.7 (4)
C7—C2—C3—C4	−0.8 (5)	N3—C36—C37—C42	144.9 (3)
C1—C2—C3—C4	177.5 (3)	C35—C36—C37—C42	−37.7 (5)
C2—C3—C4—C5	−0.8 (5)	C42—C37—C38—C39	3.3 (5)
C3—C4—C5—C6	1.3 (5)	C36—C37—C38—C39	−173.2 (3)
C3—C4—C5—C8	−179.7 (3)	C37—C38—C39—C40	−0.8 (6)
C4—C5—C6—C7	−0.1 (5)	C38—C39—C40—C41	−2.5 (6)
C8—C5—C6—C7	−179.1 (3)	C38—C39—C40—C43	171.8 (4)
C5—C6—C7—C2	−1.5 (6)	C39—C40—C41—C42	3.2 (6)
C3—C2—C7—C6	2.0 (5)	C43—C40—C41—C42	−171.0 (3)
C1—C2—C7—C6	−176.2 (3)	C40—C41—C42—C37	−0.7 (6)
C4—C5—C8—N1	−172.5 (3)	C38—C37—C42—C41	−2.6 (5)
C6—C5—C8—N1	6.5 (5)	C36—C37—C42—C41	173.9 (3)
C4—C5—C8—C9	7.5 (5)	C39—C40—C43—O8	8.3 (6)
C6—C5—C8—C9	−173.5 (3)	C41—C40—C43—O8	−177.6 (4)
N1—C8—C9—C10	−0.3 (5)	C39—C40—C43—O7	−166.1 (4)
C5—C8—C9—C10	179.6 (3)	C41—C40—C43—O7	8.0 (6)
C8—C9—C10—C11	0.6 (5)	C35—C34—C44—C48	−153.3 (3)
C8—C9—C10—C20	−177.0 (3)	C33—C34—C44—C48	26.3 (5)
C9—C10—C11—C12	−0.6 (5)	C35—C34—C44—C45	23.9 (5)
C20—C10—C11—C12	177.1 (3)	C33—C34—C44—C45	−156.5 (3)
C10—C11—C12—N1	0.2 (5)	C48—C44—C45—C46	0.4 (5)
C10—C11—C12—C13	179.4 (3)	C34—C44—C45—C46	−177.0 (3)
N1—C12—C13—C14	−156.4 (3)	C44—C45—C46—N4	−0.2 (6)
C11—C12—C13—C14	24.4 (5)	N4—C47—C48—C44	0.1 (6)
N1—C12—C13—C18	22.0 (5)	C45—C44—C48—C47	−0.3 (5)
C11—C12—C13—C18	−157.2 (3)	C34—C44—C48—C47	177.0 (3)
C18—C13—C14—C15	0.3 (5)	C11—C12—N1—C8	0.1 (5)
C12—C13—C14—C15	178.7 (3)	C13—C12—N1—C8	−179.1 (3)
C13—C14—C15—C16	−1.2 (5)	C9—C8—N1—C12	−0.1 (5)
C14—C15—C16—C17	0.7 (5)	C5—C8—N1—C12	−180.0 (3)
C14—C15—C16—C19	−179.4 (3)	C21—C22—N2—C23	1.3 (6)
C15—C16—C17—C18	0.7 (5)	C24—C23—N2—C22	0.0 (6)
C19—C16—C17—C18	−179.2 (3)	C35—C36—N3—C32	1.4 (5)
C16—C17—C18—C13	−1.6 (5)	C37—C36—N3—C32	178.7 (3)
C14—C13—C18—C17	1.1 (5)	C33—C32—N3—C36	−0.4 (5)
C12—C13—C18—C17	−177.3 (3)	C29—C32—N3—C36	179.3 (3)
C15—C16—C19—O4	−24.3 (5)	C45—C46—N4—C47	0.0 (5)
C17—C16—C19—O4	155.6 (3)	C45—C46—N4—Mn1	−178.9 (3)
C15—C16—C19—O3	158.1 (3)	C48—C47—N4—C46	0.1 (6)
C17—C16—C19—O3	−22.0 (5)	C48—C47—N4—Mn1	178.9 (3)
C9—C10—C20—C24	129.8 (4)	O1—C1—O2—Mn1	33.0 (6)
C11—C10—C20—C24	−47.8 (5)	C2—C1—O2—Mn1	−147.3 (3)
C9—C10—C20—C21	−47.7 (5)	O3—C19—O4—Mn1^ii^	−88.5 (9)
C11—C10—C20—C21	134.7 (4)	C16—C19—O4—Mn1^ii^	94.1 (8)
C24—C20—C21—C22	−1.0 (5)	O6—C25—O5—Mn1^i^	−6.6 (4)
C10—C20—C21—C22	176.6 (3)	C26—C25—O5—Mn1^i^	172.2 (3)
C20—C21—C22—N2	−0.8 (6)	O5—C25—O6—Mn1^i^	7.0 (4)
N2—C23—C24—C20	−1.8 (5)	C26—C25—O6—Mn1^i^	−171.7 (3)
C21—C20—C24—C23	2.3 (5)	O8—C43—O7—Mn1^iii^	−3.5 (6)
C10—C20—C24—C23	−175.3 (3)	C40—C43—O7—Mn1^iii^	170.8 (3)
O6—C25—C26—C31	175.4 (3)	O7—C43—O8—Mn1^iii^	3.1 (5)
O5—C25—C26—C31	−3.4 (5)	C40—C43—O8—Mn1^iii^	−171.2 (3)
O6—C25—C26—C27	−3.0 (5)	C46—N4—Mn1—O4^iv^	29.1 (3)
O5—C25—C26—C27	178.3 (3)	C47—N4—Mn1—O4^iv^	−149.7 (3)
C31—C26—C27—C28	−0.7 (5)	C46—N4—Mn1—O7^v^	116.5 (3)
C25—C26—C27—C28	177.7 (3)	C47—N4—Mn1—O7^v^	−62.3 (3)
C26—C27—C28—C29	−0.4 (5)	C46—N4—Mn1—O6^vi^	−130.6 (3)
C27—C28—C29—C30	0.9 (5)	C47—N4—Mn1—O6^vi^	50.6 (3)
C27—C28—C29—C32	178.8 (3)	C46—N4—Mn1—O5^vi^	−87.9 (3)
C28—C29—C30—C31	−0.2 (5)	C47—N4—Mn1—O5^vi^	93.3 (3)
C32—C29—C30—C31	−178.1 (3)	C46—N4—Mn1—O2	−49.5 (3)
C29—C30—C31—C26	−0.9 (5)	C47—N4—Mn1—O2	131.7 (3)
C27—C26—C31—C30	1.4 (5)	C46—N4—Mn1—O8^v^	155.6 (3)
C25—C26—C31—C30	−177.0 (3)	C47—N4—Mn1—O8^v^	−23.2 (3)
C28—C29—C32—N3	−16.8 (5)	C46—N4—Mn1—C25^vi^	−119.0 (3)
C30—C29—C32—N3	161.0 (3)	C47—N4—Mn1—C25^vi^	62.3 (3)
C28—C29—C32—C33	162.8 (3)	C1—O2—Mn1—O4^iv^	61.8 (3)
C30—C29—C32—C33	−19.3 (5)	C1—O2—Mn1—O7^v^	7.9 (5)
N3—C32—C33—C34	−1.4 (5)	C1—O2—Mn1—N4	159.3 (3)
C29—C32—C33—C34	178.9 (3)	C1—O2—Mn1—O6^vi^	−95.9 (3)
C32—C33—C34—C35	2.2 (5)	C1—O2—Mn1—O5^vi^	−39.0 (3)
C32—C33—C34—C44	−177.3 (3)	C1—O2—Mn1—O8^v^	−145.8 (3)
C33—C34—C35—C36	−1.3 (5)	C1—O2—Mn1—C25^vi^	−68.1 (3)
C44—C34—C35—C36	178.2 (3)		

Symmetry codes: (i) *x*+1/2, −*y*+1/2, *z*+1/2; (ii) *x*, −*y*−1, *z*−1/2; (iii) *x*+1/2, *y*−1/2, *z*; (iv) *x*, −*y*−1, *z*+1/2; (v) *x*−1/2, *y*+1/2, *z*; (vi) *x*−1/2, −*y*+1/2, *z*−1/2.

### Hydrogen-bond geometry (Å, º)

**Table:**

*D*—H···*A*	*D*—H	H···*A*	*D*···*A*	*D*—H···*A*
N2—H2*A*···O3^vii^	0.89 (2)	1.67 (2)	2.548 (4)	169 (5)
O1—H1*A*···O5^vi^	0.88 (2)	1.77 (3)	2.612 (4)	159 (6)
O1*W*—H1*W*···O3	0.84	2.12	2.963 (6)	180

Symmetry codes: (vi) *x*−1/2, −*y*+1/2, *z*−1/2; (vii) *x*+1/2, −*y*−3/2, *z*+1/2.
