# Supplementary figures and images for: Crystal structure of poly[[μ3-4,4′-(4,4′-bipyridine-2,6-diyl)dibenzoato]{μ2-4-[6-(4-carboxyphenyl)-4,4′-bipyridin-4′-ium-2-yl]benzoato}manganese(II)] hemi­hydrate]
Source: Acta Crystallogr Sect E Struct Rep Online. 2014 Oct 24;70(Pt 11):m374–5. doi: 10.1107/S160053681402279X (PMC4257299; doi:10.1107/S160053681402279X)

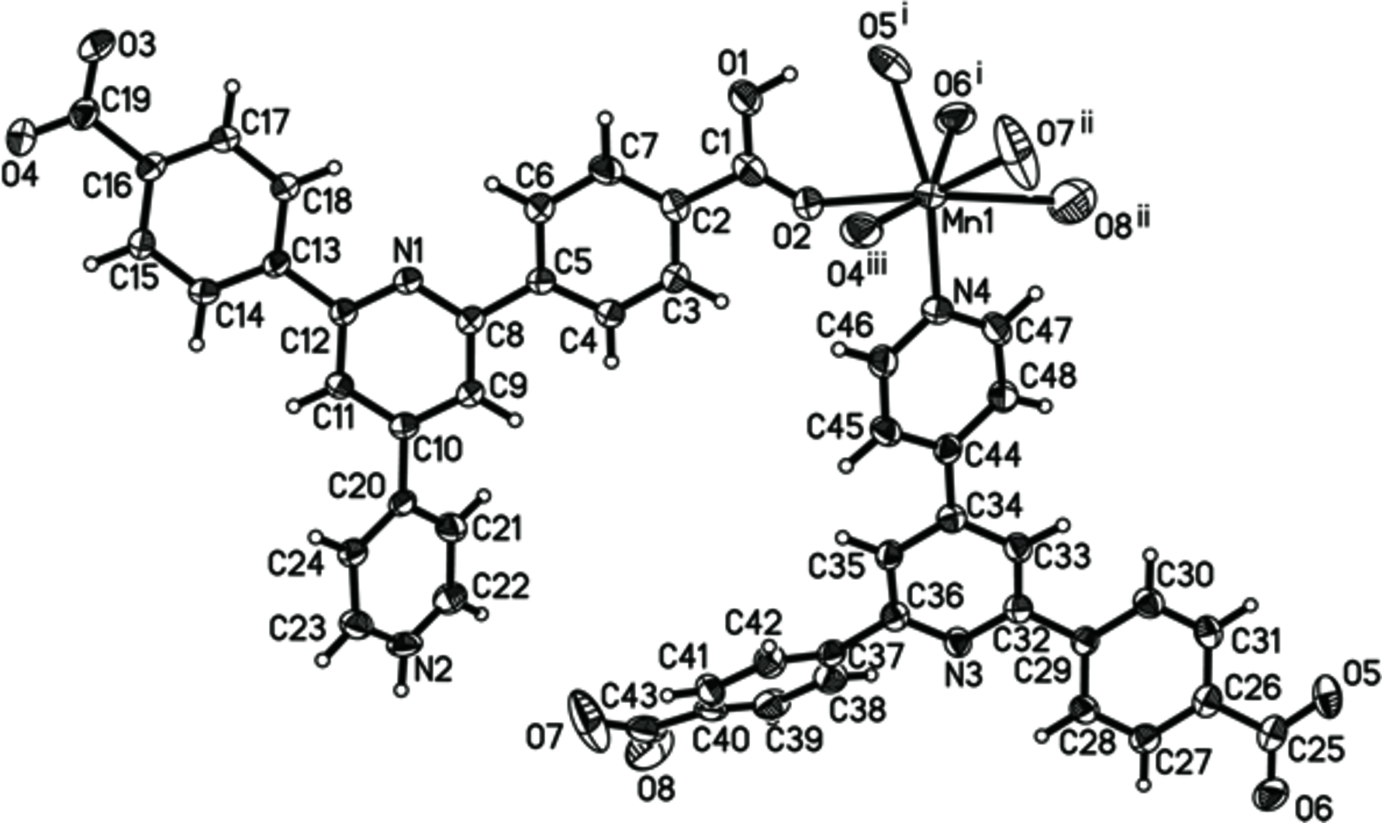

Supplement: Supplementary file 3 [file e-70-0m374-fig1.tif]

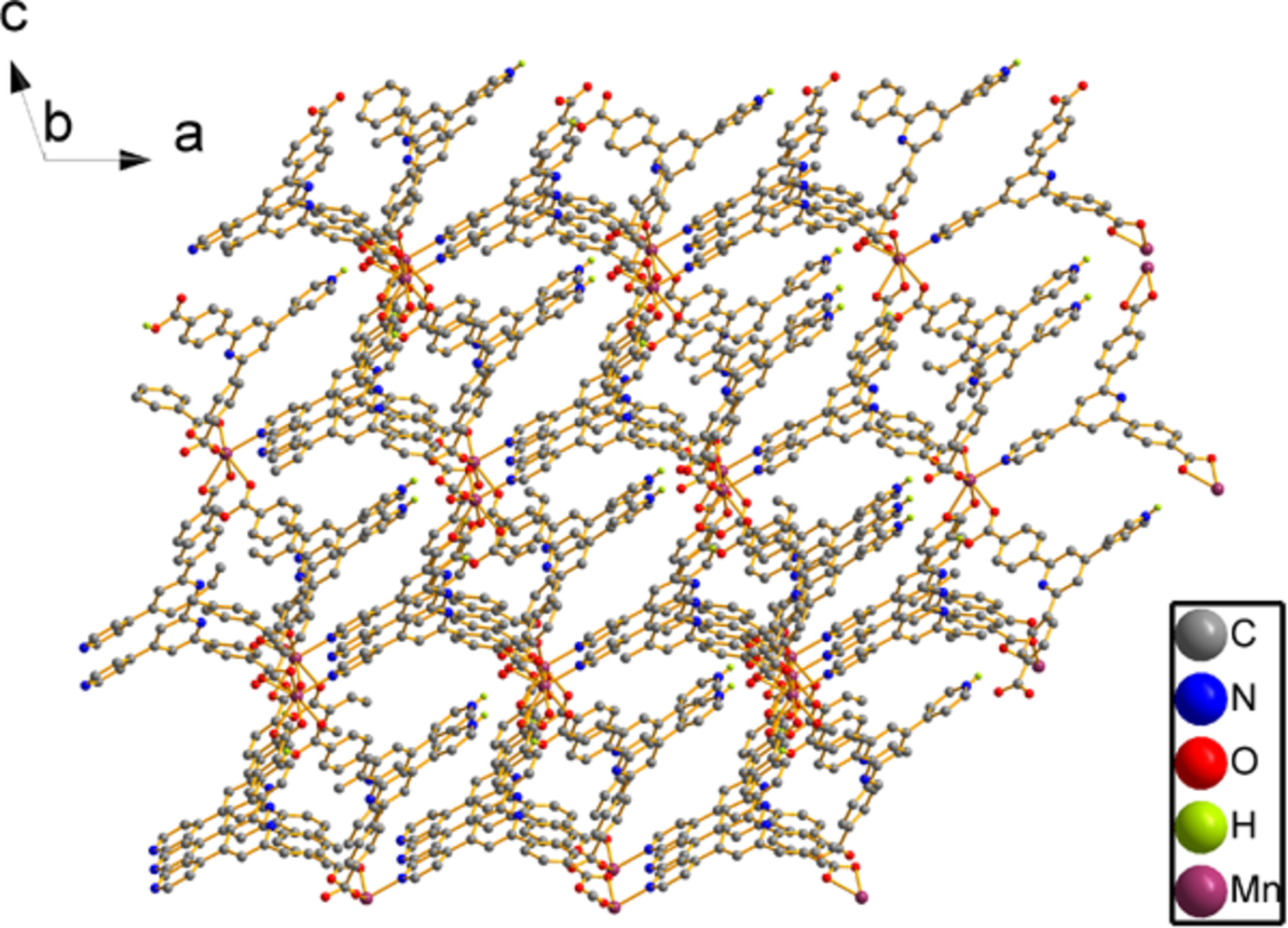

Supplement: Supplementary file 4 [file e-70-0m374-fig2.tif]
